# The impacts of climate change on violent conflict risk: a review of causal pathways

**DOI:** 10.1088/2515-7620/ad8a21

**Published:** 2024-11-11

**Authors:** Xiaolan Xie, Mengmeng Hao, Fangyu Ding, Jürgen Scheffran, Tobias Ide, Jean-François Maystadt, Yushu Qian, Qian Wang, Shuai Chen, Jiajie Wu, Kai Sun, Tian Ma, Dong Jiang

**Affiliations:** 1 School of Geography & Environmental Science,Guizhou Normal University, Guiyang, 550001, People’s Republic of China; 2 Institute of Geographic Sciences and Natural Resources Research, Chinese Academy of Sciences, Beijing 100101, People’s Republic of China; 3 College of Resources and Environment, University of Chinese Academy of Sciences, Beijing 100049, People’s Republic of China; 4 Institute of Geography, Center for Earth System Research and Sustainability, University of Hamburg, Hamburg 20144, Germany; 5 Harry Butler Institute, Murdoch University, Perth, Australia; 6 IRES/LIDAM, UCLouvain, Ottignies-Louvain-la-Neuve, Belgium; 7 FNRS - Fonds de la Recherche Scientifique,Brussels, Belgium; 8 Department of Economics, Lancaster University Management School, Lancaster, United Kingdom; 9 Centre for Tropical Medicine, Nuffield Department of Clinical Medicine, University of Oxford, United Kingdom; 10 GeoAI Lab, Department of Geography, University at Buffalo, Buffalo, NY, United States of America

**Keywords:** climate change, violent conflict risk, causal pathways

## Abstract

The potential impacts of climate change on violent conflict are high on the agenda of scholars and policy makers. This article reviews existing literature to clarify the relationship between climate change and conflict risk, focusing on the roles of temperature and precipitation. While some debate remains, substantial evidence shows that climate change increases conflict risk under specific conditions. We examine four key pathways through which climate affects conflict: (i) economic shocks, (ii), agricultural decline, (iii) natural resources competition, and (iv) migration. Key gaps include limited long-term data, insufficient integrated studies, and the inadequate understanding of causal mechanisms, necessitating transdisciplinary research that addresses social vulnerability and underlying pathways.

## 1. Introduction

Violent conflict has been and will remain a serious global issue despite the commitment of the United Nations to promote peace through achieving Sustainable Development Goals (SDGs), in particular SDG #16 (Nations 2015). According to the Geo-referenced Event Dataset (GED) (version 20.1) of the Uppsala Conflict Data Program’s (UCDP) database statistics, there were estimated to be more than 2.86 million deaths from 1989 to 2021 (Pettersson *et al* 2021) due to armed conflict. Such conflicts of a violent nature may endanger lives and cause considerable damage, and though heterogeneous in nature, can be driven by similar risk factors (Trinn and Wencker 2021).

In previous research, some studies suggested that conflict risk is associated with climate change, particularly in countries or regions highly dependent on agriculture for income and food production (von Uexkull *et al* 2016, Ide *et al* 2020). Others showed a higher probability that in cases in which social capital for adaptation is limited and society is more sensitive to climate change, such greater vulnerability leads to a higher probability of climate shocks translating into conflict risks (Buhaug and von Uexkull 2021). Such societies may be locked in a vicious circle that traps them in conflict, vulnerability, and climate change impacts (Buhaug and von Uexkull 2021). It is thus imperative to clarify and understand the impacts of climate change on conflict risk, especially in currently vulnerable regions, to design policies responding to conflict and mitigating future conflict risk.

Research on the climate-conflict link increasingly emerged around the year 2007 and rapidly gained traction in the last decade. According to some authors, there are over 1,000 studies in the broader research field (Sharifi *et al* 2021). A number of review articles take stock of and provide an overview about research on climate change and conflicts. Some of these reviews focus on particular aspects, such as research methods (Selby and Hoffmann 2014, Ide 2017), expert opinions (Mach *et al* 2019), or blind spots of the research field (Adams *et al* 2018, Scartozzi 2021). Several other review articles deal with evidence on a climate-conflict nexus more broadly (Scheffran *et al* 2012, Hsiang *et al* 2013, Koubi 2019). Meanwhile, research on the topic has evolved rapidly, even since the last IPCC report in early 2022 (Ide 2023, Koren and Schon 2023, Michelini *et al* 2023, von Uexkull *et al* 2023).

This article contributes to the ongoing discourse by offering a comprehensive review of the latest evidence on climate change and conflict risk, focusing specifically on temperature and precipitation. In contrast to previous reviews, we further explore the potential causal pathways—economic shocks, agricultural decline, resource competition, and migration—that link climate change to violent conflict (Koubi 2019, van Baalen 2021). Our primary objective is to clarify these pathways and identify research priorities to deepen understanding of the climate-conflict relationship.

The literature reviewed in this paper was gathered from databases such as Scopus and Web of Science using search terms related to climate change and violent conflict. We focused on violent conflict within states—such as civil wars, community violence, and riots—as the literature suggests that climate-related interstate conflict is less likely (O’Loughlin *et al* 2014, Mach *et al* 2019, Helman *et al* 2020).

## 2. Is there a significant impact of climate change on conflict risk?

A large and increasing number of studies have explored the potential causal links between climate change and conflict, mainly focusing on short-term climate variability related to temperature and precipitation. While the latter are affected by long-term climate changes, we discuss the need to focus more on the conflict implications of climatic changes (rather than variability) below.

### 2.1. The impact of temperature on conflict risk

Research on the linkage between temperature and conflict has been conducted on various scales. At the individual level, psychological or physiological links have been claimed between temperature and conflict (Miles-Novelo and Anderson 2019). Psychologists and sociologists have examined how uncomfortable temperatures could affect the thoughts, emotions, and aggressive behaviors of individuals, indicating that heat might have a negative effect and inspire violent feelings (Anderson *et al* 2000). Several psychological theories offered interpretations of such results. For example, the general aggression model (GAM) stated that the stimulation from the environment (e.g. temperature) could raise levels of individual irritability and thus increase their aggressiveness (DeWall *et al* 2011). The prominent routine activity theory (RAT) proposed that high temperatures would likely increase the frequency of interactions between people, thereby raising the chance of interpersonal violence (Anderson and Bushman 2002).

To verify whether these phenomena observed in micro settings would translate to a larger level and to inter-group conflict, scholars have conducted several studies. On the global scale, a few analyses showed that rising temperatures are associated with the increasing probability of conflict (Ge *et al* 2022). On the regional scale, some researchers have found a similar association between higher temperatures and various forms of conflict risks (Wang *et al* 2022). Examples include Burke *et al* (2009) for sub-Saharan Africa and Hsiang *et al* (2011) for countries affected by El Nino-La Nina cycles. These findings were, however, heavily contested (Buhaug 2010, Buhaug *et al* 2014). Other studies also suggested rather limited impacts of temperature on the risk of various types of conflict (Bernauer *et al* 2012, Klomp and Bulte 2013, Yeeles 2015).

In addition to these debates, it is important to consider the varying levels of temperature increases and their differential impacts on conflict. While moderate temperature rises have been linked to increased interpersonal violence, such as violent crime, extreme temperature events—like heatwaves—might lead to larger social disruptions or intensify resource-driven conflicts between groups (Hsiang *et al* 2013). However, identifying the specific thresholds or tipping points where temperature increases significantly escalate conflict remains an ongoing challenge and an important area for further research (Scheffran *et al* 2012).

In recent years, more nuanced datasets with a higher spatial and temporal resolution facilitated a new series of studies (Thalheimer *et al* 2023, Guo *et al* 2024), several of which find an effect of higher temperatures on conflict risks, for instance in tropical regions (Wang *et al* 2022), Asia (Hao *et al* 2022), or Africa and the Middle East (Helman *et al* 2020, Abdi *et al* 2023). However, the substantial effect is often small. Furthermore, validating the effects of temperature on conflict risks via process tracing and specific pathways remains challenging. This makes it difficult to substantiate the statistical signal with clear causal evidence of a link between heat and conflict, and further strengthens the need to focus on causal pathways underlying the climate-conflict nexus (see section 3).

### 2.2. The impact of precipitation on conflict risk

Similarly, research on the effects of precipitation on conflict has evolved significantly in recent years. Just as for temperature, early scholarship was deeply divided. Several analyses found that droughts increase resource scarcity, dampen economic growth, worsen food insecurity, and are hence associated with a higher likelihood of violent conflict (Maystadt & Ecker 2014, von Uexkull 2014, Maystadt *et al* 2015, Raleigh *et al* 2015). Yet, other analyses failed to confirm such a link (Theisen *et al* 2011, Couttenier and Soubeyran 2014, Wischnath and Buhaug 2014, Yeeles 2015).

Recent studies are more moderate and nuanced in their conclusions (Damette and Goutte 2023, Karesdotter *et al* 2023, Petit *et al* 2023). There is an increasing scholarly consensus that droughts and precipitation declines enhance the risks of various forms of conflict, with a generally stronger impact on communal violence and riots than on high-intensity civil wars (Unfried *et al* 2022). However, climate-related precipitation declines are not the most important conflict drivers, and a drought-conflict nexus only manifests if a number of contextual factors are present. The latter include the absence of wells, dams, and irrigation infrastructures (Detges 2016, Mary 2022), agricultural dependence and ethnic discrimination (von Uexkull *et al* 2016), and pre-existing grievances and a lack of proper state action (Ide *et al* 2021), among others. The presence of a drought-conflict nexus is further indicated by micro-level evidence suggesting that during droughts, altruism decreases (Döring and Hall 2023), outgroup hostility is more explicit (Chung and Rhee 2022), and people are more likely to support the use of political violence (Detges 2017, von Uexkull *et al* 2023).

Two important qualifications are due here. First, increased precipitation can also make violent conflict more likely in certain settings. It allows rebels to live off the land and feed their fighters (Schon *et al* 2023b), can trigger the targeting of rich agricultural areas by state forces (Selby and Hoffmann 2014, Koren and Schon 2023), or allow cattle raiders to hide in dense vegetation while their tracks are washed away (Adano *et al* 2012). Furthermore, climate change is also predicted to increase rainfall in some regions (IPCC 2022), potentially even leading to floods (see section 2.3).

Second, just because drought makes conflict onset or incidence more likely, this does not mean that there are deterministic linkages between droughts and conflicts. There have been very strong claims, for instance, about droughts driving the onset of the Syrian civil war (Kelley *et al* 2015) or political instability around Lake Chad. Studies like those by Selby *et al* (2017), Daoudy (2020), Selby *et al* (2022), and Daoust and Selby (2022) played an important role in debunking and nuancing such claims about droughts and violent conflict. Recent evidence suggests, however, that unusually low rainfall was at least one conflict driver (interacting with others, presumably more important) both in Syria (Ash and Obradovich 2020, Dinc and Eklund 2023) and Western Africa (Newman *et al* 2023).

### 2.3. The impacts of weather extremes on conflict risks

There is considerable evidence that climate change increases the frequency of extreme weather events like storms, floods, heatwaves, landslides, or droughts (the latter is discussed in section 2.2). When such hazards hit vulnerable societies, they often have disastrous consequences in terms of deaths and destruction (Boccard 2021).

Researchers have long-studied the impacts of climate-related disasters on violent conflict risks. Initially, the results were inconclusive and contradictory. For instance, Nel and Righarts (2008) and Berrebi and Ostwald (2011) find that disasters increase the likelihood of armed conflict onset and terrorist attacks. The results of Nardulli *et al* (2013) are more mixed, while Omelicheva (2011) and Slettebak (2012) conclude that there is no evidence for a disaster-conflict nexus.

In recent years, more consistent evidence has appeared (Dinc and Eklund 2024, Mitchell 2024). Scholars now mostly agree that climate-related disasters increase violent conflict risks, but only under certain circumstances. Both Walch (2018) and Ide (2023), for instance, point out that when disasters negatively affect conflict parties, fighting is unlikely to escalate, and rather tends to de-escalate in the short-term. However, if the disaster weakens the state or benefits of a rebel group, the latter tend to upscale their violent efforts. In line with this, but focusing on pro-state actors, Eastin and Zech (2022) present evidence that disaster-induced poverty facilitates recruitment campaigns by community militias in the Philippines.

Going beyond conflict intensity, research by Schleussner *et al* (2016) and Ide *et al* (2020) finds that climate-related disasters like floods or storms increase the risk of armed conflict incidence and onset if certain conditions are present, such as ethnic exclusion, ethnic heterogeneity, and low levels of human development. There is also ample evidence of protests and riots occurring after governments mishandling disaster preparation and relief (Ide *et al* 2021, Petrova 2022). Climate-related disasters can also prolong civil wars if they give the conflict parties time and opportunities to regroup or if inflowing aid is diverted for military purposes (Eastin 2016). However, evidence on a disaster-conflict duration link is limited and does not hold when, for instance, disasters destroy the resource base of the rebels (Tominaga and Lee 2021).

## 3. How does climate change affect conflict risk?

The above results show that a majority of experts and studies now agree that climate change increases violent conflict risks, even though it is usually not the most important conflict driver. Rather, climate change amplifies existing conflict risks in contexts already prone to violent confrontations. In line with this, several older studies looking for a general association between climate and conflict were unable to detect a relationship (Buhaug 2010, Bernauer *et al* 2012). However, many newer studies using more fine-grained data and focusing on relevant context factors have supported the existence of a climate-conflict nexus (von Uexkull *et al* 2016, Koubi *et al* 2021, Ide 2023).

**Figure 1.**
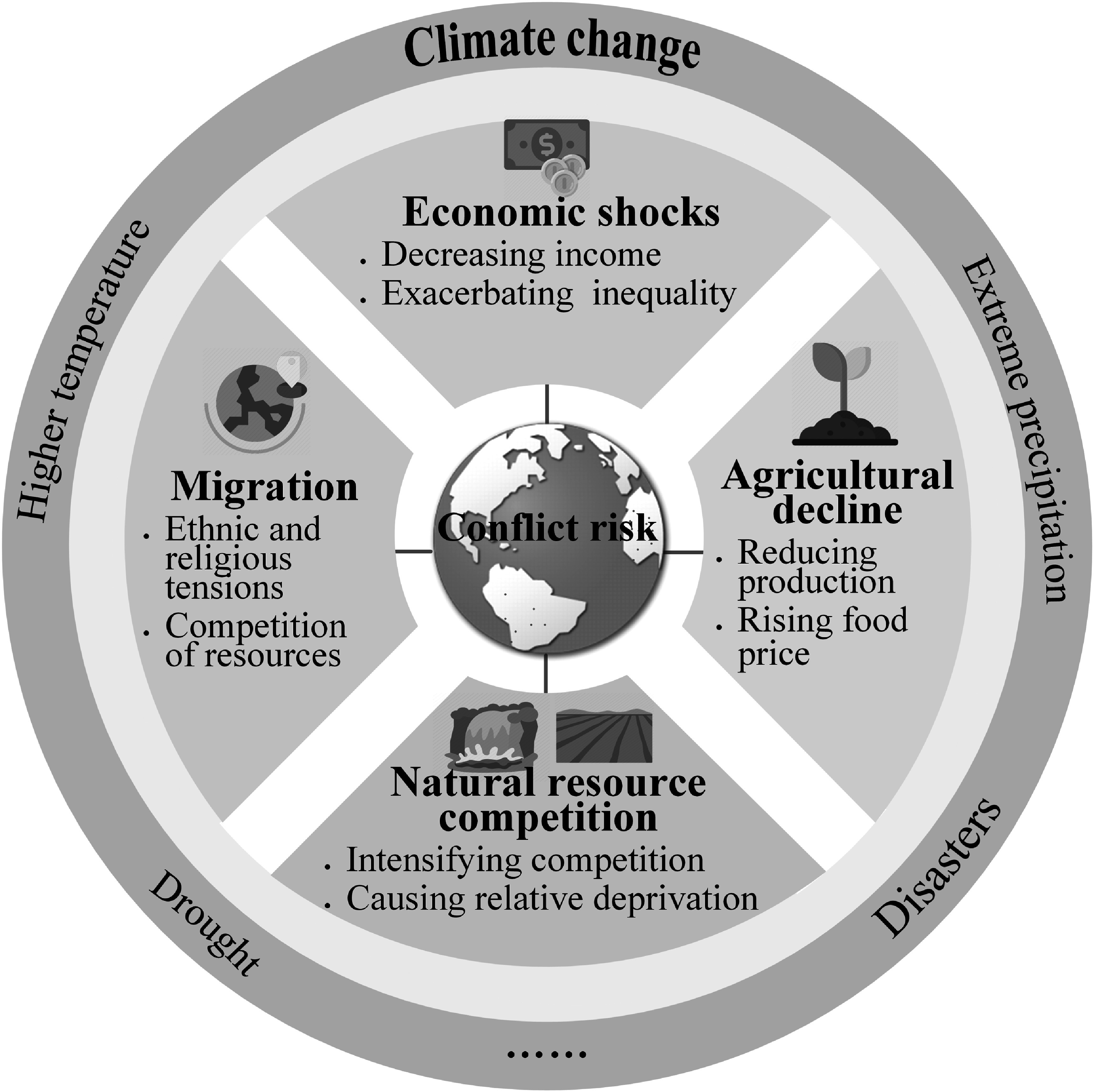
The impacts of climate change (e.g., higher temperature, drought, extreme precipitation, and disasters) on conflict risk can be amplified through economic shocks, agricultural decline, natural resources competition, and migration.

In this section, we focus on four key pathways that link climate change to conflict risk, drawing on our extensive review of the literature and the relevant causal pathways attracting the most attention (figure 1): (i) economic shocks, (ii) agricultural decline, (iii) natural resource competition, and (iv) migration. Synthesizing relevant causal pathways is important for at least three reasons. Firstly, as highlighted by Mach *et al* (2019), ‘the mechanisms of climate–conflict linkages remain a key uncertainty’. Secondly, a more explicit consideration of causal pathways allows future studies to better specify the context factors that are conductive to the emergence (or absence) of climate-conflict links. Thirdly, understanding not only whether and when, but also how climate change impacts conflict risks is crucial for designing adequate policy responses (Abrahams 2020).

Before we proceed, it is important to note that the pathways to conflict discussed here can also be triggered by non-climate-related events (e.g., pandemics can trigger economic shocks, and war can result in large-scale migration). Here, we focus specifically on how these pathways have been discussed (and the corresponding evidence produced) in the climate security literature. Furthermore, the pathways can overlap and interact to some degree, and can hence amplify or dampen each other (Scheffran *et al* 2012).

### 3.1. Economic shocks

Economic changes constitute one of the most important mediators that link climate change to conflict risk. Climate change, especially in the form of higher temperatures, lower precipitation, or changes in the intensity and frequency of extreme weather events, might affect agricultural productivity, economic growth, and development (Burke *et al* 2015, Zhang *et al* 2024). Consequently, the declining economic activity would exacerbate inequality and destabilize the state, as well as reducing the opportunity cost of participating in a rebellion, which are all incentives for conflict.

More specifically, when economic shocks induced by climate change cut deeply into personal income from legal production (e.g. planting corn and wheat), the opportunity cost of joining an ongoing conflict thus becomes lower (Wischnath and Buhaug 2014). For example, a severe drought devastated agricultural production in Syria, leading to significant rural distress and mass migration to urban areas. This contributed to social tensions and played a role in the outbreak of the civil war (Kelley *et al* 2015). In such situations, participation in conflict is rated more attractive when individuals (especially low-income groups) expect to earn more from criminal or insurgent activities than from lawful and peaceful ones (Chassang and Padro i Miquel 2009, Koubi *et al* 2012). Several researchers tested this argument using different methods. Miguel *et al* (2004) found that decreased rainfall can reduce national economic growth and hence increase the likelihood of civil war onset. Maystadt and Ecker (2014) showed that the reduction of income in the livestock sector is associated with an increased incentive to participate in violent conflicts. Burke and Leigh (2010) and Brückner and Ciccone (2012) demonstrated that output contraction caused by adverse weather shocks creates opportunities for democratic regime change, as seen during the Arab Spring, where economic downturns fueled popular uprisings across the region. A few other studies have also found similar results when examining the links of climate change with civil conflict onset (Burke *et al* 2009, Ide *et al* 2020), ethnic riots (Bohlken and Sergenti 2010), and various types of conflict (Hendrix and Salehyan 2012).

Further research indicated that inequality can exacerbate the adverse effects of climate change on conflict (Gupta *et al* 2023). When climate change affects the economy, it is unlikely to equally affect all individual/household incomes within a country (Canavan and Ide 2024). Naturally, less developed regions or households would suffer economically from climate change much more than rich regions or households (Moore and Diaz 2015, Lomborg 2020). The state might also prefer some groups in its response to climate and economic crises over others. Therefore, climate-driven economic downturns might amplify income and social inequalities (Ujunwa *et al* 2021), putting the poor populations in an increasingly vulnerable situation and raising their grievances. The resulting polarization might incentivize these individuals/groups to seize political power by force to redistribute wealth in their favor, thus increasing the likelihood of conflict (Cederman *et al* 2011, Guariso and Rogall 2017, Koubi 2017).

Besides the rebel groups incentivized by higher recruitment potential during economic downturns, the undermined capacity of the state to mediate conflicts and guarantee public income support, food aid, employment, and human security would also give way to increasing conflict at the communal and individual levels, including criminal and gender-based violence (Kim 2016, van Daalen *et al* 2022). Empirical research pointed out that climate-related disasters could powerfully destabilize society by reducing the state’s resilience and increasing grievances (Carmona 2024). For instance, Hurricane Katrina in the U.S. show cased how a natural disaster strained public institutions and led to significant social unrest (Nicholls and Picou 2016). When experiencing such disasters, weak regimes or social systems will be financially strained through the loss of tax revenues and foreign exchange earnings during the recession caused by weather shocks (Hendrix and Salehyan 2012, Damette and Goutte 2023). The fear, insecurity, scarcity of resources, reduced social welfare, and paralyzed public institutions could lead to various forms of dissatisfaction, eventually leading to violent actions (Nardulli *et al* 2015, Linke *et al* 2018) and non-violent protests (Ide *et al* 2021).

In summary, the literature indicates that climate change-induced loss in economic productivity increases the risk of violent conflicts within states, particularly in societies characterized by inequality and political polarization. Key arguments underlying this causal mechanism are enhanced recruitment opportunities for armed groups, state weakness, and societal grievances.

### 3.2. Agricultural decline

Agriculture is primarily affected by climate through its impacts on productivity, crop yields, arable land, and water (Schlenker and Roberts 2009, Hsiang 2010, Chen *et al* 2024). It is estimated that fluctuations in seasonal temperature and precipitation levels account for roughly a third of the variation in major global crop yields (Ray *et al* 2015). Consequently, loss of agricultural income and food insecurity caused by adverse climatic conditions could increase social grievances, providing motives or lowering the opportunity costs for engaging in rebellion (Maxwell *et al* 2010, Wischnath and Buhaug 2014). Regarding the impact of climate change on conflict working through agriculture, we found evidence supporting the relationship between climate-induced reduction in agricultural production and conflict in historical research. For example, Zhang *et al* (2007) and Jun and Sethi (2021) showed that climate-induced food shortages increased the risk of conflict when climate variation undermined agricultural production in agriculture-dominated societies and economies. Recent findings were also in line with the historical evidence, especially in areas highly dependent on rain-fed agriculture, with social divisions, and with limited coping capacities (Jun 2017, Crost *et al* 2018). Empirical research regarding the Philippines showed that excessive precipitation had hugely impacted the agricultural income in acutely deprived areas and thereby increased conflict participation (Crost *et al* 2018, Eastin 2018). Similarly, rainfall shocks could also increase the intensity of India’s Maoist insurgency via their effect on agricultural output (Gawande *et al* 2017, Fetzer 2020). For instance, the 2015 drought in Maharashtra led to widespread farmer suicides and heightened tensions in the region, illustrating how climate-induced agricultural distress can exacerbate social unrest (Kulkarni *et al* 2016). There was also research suggesting that temperature and precipitation anomalies during the core months of the crop-growing seasons could reduce the yield of rice in Indonesia (Caruso *et al* 2016), maize in sub-Saharan Africa (Jun 2017), and main crops in the Philippines (Crost *et al* 2018), resulting in the increased incidence of civil conflict.

Apart from directly affecting crop yields, climate change could also impact food prices by fluctuating the demand and supply of the affected crops, especially when the climate becomes variable (Bradbear and Friel 2013, Cater and Lew 2018, Mohamed *et al* 2024). This adds to several other factors increasing food prices, such as oil price variability, speculations on globalized food markets, and increasing investments in land for food and energy production. Rendering staple foods unaffordable for the masses increases the sense of relative deprivation, arouses new and pre-existing grievances, and further provides a political trigger with a higher risk of protests, riots, and violent collective action (Bellemare 2015, Hendrix and Haggard 2015, Jones *et al* 2017, Ten Brink *et al* 2023). Evidence favoring such theories can be found in some research in Africa, revealing the complex relationship between climate change, rising food prices, and conflict (Sharma *et al* 2024). For example, the African food riots during 2007–2008 (Berazneva and Lee 2013) and the Arab Spring (Maystadt *et al* 2014, Soffiantini 2020) have been considered partly as a consequence of price increases. Smith (2014) found that the probability of civil unrest rose with the increase in domestic food prices by using international food commodity prices and rainfall scarcity as instrumental variables. Raleigh *et al* (2015) suggested that rainfall exerts an indirect effect on conflict through its impact on food prices. Another example was food riots in countries like Mozambique and Egypt that were associated with high grain prices, following drought and its associated heat wave over Russia and severe flooding in Asia (Hunt *et al* 2021). Further studies have also identified the rise in food prices as a possible explanatory mechanism linking climate change and conflict, given the positive relationship between soaring food prices (caused by climate shocks) and social unrest (Bellemare 2015, Rudolfsen 2023).

In summary, a considerable number of studies indicate that climate change’s adverse impacts on agricultural productivity and food security could increase intrastate violent conflict risks. Grievances about livelihood loss and higher food prices as well as reduced opportunity costs for joining insurgents, are key underlying mechanisms identified in the literature. Specific cases, such as the the riots in Mozambique and Africa, illustrate how climate-related agricultural challenges can catalyze conflict (Berazneva and Lee 2013). This causal mechanism is most likely to occur in poor, agriculturally dependent, and already food insecure countries.

### 3.3. Natural resource competition

Climate change, associated with water stress and temperature change, might induce reallocation or reduction of natural resources (i.e., water and land) needed to sustain human life, and therefore induce a mismatch between supply and demand. As a consequence, natural resource reduction or allocation could trigger an agricultural collapse, increase food prices, slow economic growth, etc, which all could result in relative deprivation, and thus intensify competition over increasingly scarce resources (IPCC 2014). This is an acute issue, particularly in agriculturally dependent communities and politically fractionalized societies when they are trapped in fragile contexts characterized by demographic pressures and economic insecurities (Kelley *et al* 2015), where increasing social discontent and encouraged rebellions against the government might eventually lead to civil conflict and social unrest (Vesco *et al* 2020, Schon *et al* 2023a). Recent changes in natural resource laws and the weakness of traditional conflict resolution mechanisms are also important mediating factors (Tubi and Feitelson 2016).

Thus far, research has mainly focused on the relationships linking water and land resources to conflict, especially inter-communal violence and civil conflict, given the necessity of these resources that sustain the lives of farmers and pastoralists (Prediger *et al* 2014, Mertz *et al* 2016, Gleick and Shimabuku 2023, Lu and Yamazaki 2023). For instance, by studying 79 conflict cases in Bangladesh and Nepal, Sultana *et al* (2019) found that droughts and floods have caused shortages and imbalances in water, which directly exacerbated conflicts over resources. This is illustrated by the drought in Nepal, where farmers in the region faced severe water shortages, leading to violent clashes between agricultural communities over limited irrigation supplies. Likewise, Ide (2015) showed that the scarcity of land, water, fish, and forest resources (all of which are climate-sensitive) could trigger violent communal conflict around the world under certain circumstances. In Nigeria and Mali, for example, climate change, through increasing drought and desertification, heightens competition for limited resources such as land and water, fueling conflicts and causing displacement (Lenshie *et al* 2022). Studies of African and Middle East communities have also reported evidence about water resources transmitting climate change effects on community conflict (conflict between different farmers, pastoralists, and fisher communities) (Gleick 2014, Landis *et al* 2017, Bukari *et al* 2018, Spijkers *et al* 2021). On the other hand, it is worth noting that resource scarcity caused by climate change does not always incite conflict. Instead, in some cases, it might provide potential opportunities for cooperation and peace (Doring 2020, Johnson *et al* 2021). On the international level, water cooperation is also more prevalent than violent competition (Karesdotter *et al* 2023).

In summary, earlier evidence for the impact of natural resource scarcity on civil war was mixed (Koubi *et al* 2014). However, the majority of studies now agree that climate-related natural resource scarcity likely contributes to intergroup competition and amplifies local grievances, which can result in riots and community conflict if not mediated by strong local or state institutions. Further relevant context factors for such a causal link include agricultural dependence, pre-existing political polarization, and recent changes in relevant laws and policies.

### 3.4. Migration

Migration is a potential adaptation strategy to climate change. Seasonal or permanent migration has been associated with climate-driven ecological change, including lengthened droughts, increased climatic disasters that exacerbated unfitness for habitation, and land degradation (Thiede *et al* 2016, Dallmann and Millock 2017, Kwanhi *et al* 2024, Raimi *et al* 2024), and decreased access to natural capital (McMichael *et al* 2012). Although poor infrastructures, scarcity of livelihood opportunities, and credit constraints still limit the ability of the poorest to migrate as an adaptation strategy (Gray and Mueller 2012, Beine and Parsons 2015, Cattaneo and Peri 2016, Nawrotzki and DeWaard 2018, Hunter *et al* 2022), climate change could affect migration patterns (Berlemann and Steinhardt 2017, Borderon *et al* 2019, Moore and Wesselbaum 2022, Rikani *et al* 2023).

It is argued that climate change-induced migration might promote conflict through increasing competition over resources in communities while igniting ethnic and religious tensions between migrants and domestic people in some contexts (Brzoska and Fröhlich 2016, Burrows and Kinney 2016, Wiederkehr *et al* 2022, Saraiva and Monteiro 2023). Such tensions also depend on the nature of the inter-group antagonism (Esteban *et al* 2012, Amodio and Chiovelli 2018, Bazzi *et al* 2019). For example, a survey in Kenya showed that relocation driven by drought or water shortages could lead to conflict due to labor and residential housing market competition (Linke *et al* 2018). In Sudan’s Darfur region, De Juan (2015) showed that migrations fostered by ecological changes from resource-reducing areas to more thriving neighborhoods have aggravated resource scarcity and interethnic tensions in areas of high immigration, thus escalating the risk of conflict. In northern Nigeria, climate change has degraded vegetation, prompting Fulani herders to migrate south into Christian areas, leading to violent conflicts with local farmers over resources (Okunade and Kohon 2023). Similar patterns have been found in Nigeria (Akinyemi and Olaniyan 2017) and India (Bhavnani and Lacina 2015). The group around Vally Koubi also concluded that climate-related migrants are more likely to experience conflicts and participate in social movements in their new homes (Koubi *et al* 2018, Koubi *et al* 2021). A well-known—although contested (Selby *et al* 2017)−case study is the one on Syria by Kelley *et al* (2015) where the Syrian civil war is argued to result from drought-induced migration.

On the other hand, several studies argued against the above theory and claimed that the climate change-migration nexus likely doesn’t directly lead to violent conflict. Instead, they suggested that migration might alleviate climate-induced pressure on economies and the environment by decongesting and redistributing the population to countries with higher carrying capacity without provoking social unrest (Byravan and Rajan 2017, Cattaneo and Bosetti 2017, Bosetti *et al* 2021). Cottier and Salehyan (2021) found that climate change reduces international migration because it deprives households of the resources to fund migration moves. In line with this, a recent analysis finds no impact of disaster on migration for the conflict in Bangladesh (Petrova 2021).

In summary, empirical evidence for migration as the causal mechanism between climate change and violent conflict remains ambiguous, with some studies finding a link, while other researchers remain skeptical. This indicates the requirement for additional research, which might either disregard this causal mechanism or specify context factors under which it usually occurs. More research should also be devoted to studying the risk of conflict among the immobile population.

### 3.5. Reflection

Early research showed considerable disagreement about the existence of a climate-conflict nexus. Recent studies accounting for relevant scope conditions tend to show stronger support for climate change to have effects on conflict risks. Specifying the pathways underlying the climate-conflict nexus further advances the research field and increases the policy relevant to results.

For example, by simultaneously considering the underlying pathways such as economics, agriculture, and resources that drive climate conflict, Helman *et al* (2020) quantified the direct and indirect effects of climate change on conflict risk in Africa and the Middle East. They illustrated that climate-conflict connections were complex due to multiple mechanisms working simultaneously (Helman *et al* 2020). Similarly, Xie *et al* (2022) revealed the complex link between climate change and conflict risk in South Asia. By partitioning impact channels into detailed paths, they found some pathway effects that offset each other, leading to a net-zero synthesized effect of the related factors. Likewise, Ide *et al* (2020) find that climate-related disasters increase the risks of conflict, primarily by providing opportunities for rebel groups in connection with economic decline and agricultural losses. This is observed only in situations where low levels of development and ethnic exclusion coincide. By contrast, studies find rather limited support for the migration pathway (Cottier *et al* 2021, Petrova 2021).

To better understand these dynamics, applying rigorous methodological approaches is crucial. For example, Large-N statistical analyses can effectively test for indirect and interaction effects among variables, enabling researchers to control for confounding factors and isolate the specific influence of climate on conflict. Additionally, process tracing in qualitative case studies can provide deeper insights into the causal mechanisms, allowing researchers to track the sequence of events that lead from climate change to conflict and reveal contextual influences. Mixed-methods approaches that integrate both quantitative and qualitative data further enhance our understanding of the climate-conflict nexus. Therefore, it is imperative to break down climate-conflict mechanisms, specify relevant scope conditions, and include these specifications in the research design. Incorporating a variety of methods—such as large-N statistical analyses, process tracing, and mixed-methods approaches—will strengthen causal claims and enhance our understanding of the complex relationship between climate change and conflict (see (Ide 2017) for a review specifically on methods.)

## 4. Conclusion

The potential causal links between climate change and conflict risk have attracted much scientific, public, and political attention. In this review, we have summarized the recent literature on the links between climate change and conflict, and noted that most recent studies (particularly those utilizing fine-grained data and considering relevant contextual factors) provide support for the claim that climate change increases conflict risks. In addition, we have outlined possible key pathways through which climate change affects conflict risk: economic shocks, agricultural decline, natural resource competition, and migration. These pathways help clarify the climate-conflict linkage and are essential for advancing research and policy. However, other pathways, such as political institutions, ethnic divisions, maladaptation, energy vulnerability, geopolitical rivalry, and biodiversity loss, also warrant further exploration.

Despite past research identifying potential climatic effects on conflict, more targeted investigations are needed to fully grasp the complexity of this relationship. In particular, future research should emphasize specific regions and conflict types where vulnerabilities to climate impacts are more pronounced. For example, regions with high resource dependency or geopolitical tensions, such as the Sahel, South Asia, or coastal zones threatened by sea-level rise, present critical case studies for further exploration. The role of different conflict types, ranging from inter-state disputes to communal violence, also needs further exploration to understand how climate pressures exacerbate specific forms of conflict.

In addition, varying social and geographical contexts create diverse vulnerabilities, leading to inconsistent responses to climate-induced conflict across regions (Buhaug and von Uexkull 2021). There is a need for greater attention to the concept of vulnerability, shaped by socio-economic contexts, political institutions, and historical legacies such as colonialism. Climate-conflict research should engage more with political ecology and other critical approaches (Ide *et al* 2023), which would help shift focus beyond ‘state-centric’ perspectives to examine how not only rebels or local communities but also political elites and state actors may respond violently to climate stress (Selby and Hoffmann 2014). A systematic exploration of feedback loops between climate change, vulnerability, and conflict risk is critical (Buhaug *et al* 2014).

Moreover, most prior research has primarily focused on short-term climate variability (e.g., seasonal or annual changes) rather than on the long-term shifts in climatic averages or increasing variability (van Weezel 2020). Future studies should investigate how gradual climatic shifts, such as rising temperatures or sea-level rise, influence conflict dynamics over extended periods.

Finally, future research must aim to quantify the underlying mechanisms driving the climate-conflict relationship, paying closer attention to the interactions between climate impacts and social, economic, and political drivers. Advanced methods, such as Structural equation Modeling and GIS-based risk analysis, alongside micro-level data (e.g., satellite imagery and social media), could refine our understanding of pathways that previous research has only broadly outlined (Ide 2017, Mach *et al* 2020). Researchers should also examine a broader range of climate factors beyond temperature and precipitation, such as sea-level rise, climate tipping points (Scheffran 2020, Franzke *et al* 2022), and the implications of climate actions on conflict (Dabelko *et al* 2017, Nadiruzzaman *et al* 2022, Buhaug *et al* 2023).

## Data Availability

No new data were created or analysed in this study.
